# Diagnostic Accuracy of Calcitonin, Procalcitonin, proGRP, miR-375 and FNA-Washout Calcitonin for Medullary Thyroid Carcinoma: A Systematic Review and Diagnostic Meta-Analysis

**DOI:** 10.3390/life16091456

**Published:** 2026-08-31

**Authors:** Dan Brebu, Flaviu Ionut Faur, Andrei Korodi, Mihai Cosmin Burta, Mircea Selaru, Paul Pasca, Marco Marian, Ioana Adelina Faur, Mihaela Andreea Precup, Ciprian Duta, Amadeus Dobrescu, Tudor Mateescu, Mihaela Pasca-Fenesan

**Affiliations:** 1 IInd Surgery Clinic, Timisoara Emergency County Hospital, 300723 Timisoara, Romania; brebu.dan@umft.ro (D.B.); flaviu.faur@umft.ro (F.I.F.); mihai.burta@umft.ro (M.C.B.); paul.pasca@umft.ro (P.P.); marian.marco@umft.ro (M.M.); adelina.clim@umft.ro (I.A.F.); dobrescu.amadeus@umft.ro (A.D.); 2 X Department of General Surgery, “Victor Babes” University of Medicine and Pharmacy Timisoara, 300041 Timisoara, Romania; duta.ciprian@umft.ro; 3 2nd Surgery Clinic, Emergency Clinical County Hospital of Arad, 2-4 Andreny Karoly Str., 310037 Arad, Romania; 4 Department of Medicine, Faculty of Medicine, “Vasile Goldis” Western University of Arad, L. Rebreanu St. 86, 310048 Arad, Romania; 5 Doctoral School of Medicine, “Victor Babes” University of Medicine and Pharmacy Timisoara, Eftimie Murgu Square 2, 300041 Timisoara, Romania; mihaela.precup@umft.ro; 6 IIIrd Surgery Clinic, Timisoara Emergency County Hospital, 300723 Timisoara, Romania; 7 Department of Thoracic Surgery, “Dr. Victor Babeș” Clinical Hospital of Infectious Diseases and Pneumology, Gheorghe Adam 13, 300310 Timisoara, Romania; tudor_mat@yahoo.ro; 8 Microscopic Morphology/Histology, Department of Clinical Oncology, Doctoral School in Medicine, “Victor Babes” University of Medicine and Pharmacy Timisoara, 300041 Timisoara, Romania; mihaela.fenesan@umft.ro; 9 Oncohelp Clinic, 300425 Timisoara, Romania

**Keywords:** medullary thyroid carcinoma, calcitonin, procalcitonin, miR-375, diagnostic biomarkers, thyroid nodules, diagnostic meta-analysis, SROC, diagnostic accuracy, endocrine oncology, FNA-washout calcitonin, QUADAS-2

## Abstract

Background: Medullary thyroid carcinoma (MTC) is a rare but aggressive neuroendocrine thyroid malignancy in which early diagnosis is essential for improving clinical outcomes. Although calcitonin (CT) remains the standard diagnostic biomarker, its interpretation may be limited by false-positive elevations, assay variability, and indeterminate cut-off values. Consequently, alternative biomarkers such as procalcitonin (PCT), circulating microRNA-375 (miR-375), and fine-needle aspiration washout calcitonin (FNA-CT) have gained increasing interest. Methods: A systematic review and diagnostic meta-analysis were conducted to evaluate the diagnostic performance of circulating and tissue-related biomarkers for MTC. Eligible studies reporting sufficient diagnostic accuracy data were included. Pooled sensitivity, specificity, positive likelihood ratio (LR+), negative likelihood ratio (LR−), diagnostic odds ratio (DOR), and summary receiver operating characteristic (SROC) curves were calculated using a bivariate random-effects model. Methodological quality was assessed using the QUADAS-2 tool. Results: Five diagnostic datasets were included in the quantitative synthesis. The pooled sensitivity and specificity were 0.90 (95% CI: 0.84–0.94) and 0.99 (95% CI: 0.97–0.997), respectively. The pooled LR+ was 84.78, while the pooled LR− was 0.10. The pooled diagnostic odds ratio was 857.30, indicating excellent discriminatory diagnostic performance. SROC analysis demonstrated near-perfect overall diagnostic accuracy with an area under the curve (AUC) of 0.986. Procalcitonin-based approaches showed the most consistent diagnostic performance across studies, while combined CT + PCT strategies achieved the highest overall diagnostic accuracy. Emerging biomarkers such as miR-375 and FNA-CT also demonstrated promising results, although evidence remains limited. Conclusions: Biomarker-based diagnostic strategies demonstrate excellent diagnostic accuracy for medullary thyroid carcinoma. Among the evaluated biomarkers, procalcitonin-based approaches, particularly combined CT + PCT strategies, showed the most robust and clinically relevant diagnostic performance. These findings support the potential integration of procalcitonin into future diagnostic algorithms for MTC. Further large-scale prospective studies are required to validate diagnostic thresholds and standardize biomarker assessment protocols.

## 1. Introduction

Medullary thyroid carcinoma (MTC) is a rare neuroendocrine malignancy originating from parafollicular C-cells of the thyroid gland and accounts for approximately 1–2% of all thyroid cancers [1,2,3,4]. Despite its relatively low incidence, MTC is associated with significantly higher morbidity and mortality compared with differentiated thyroid carcinomas because of its early metastatic potential and more aggressive biological behavior. Early diagnosis remains critically important, as survival outcomes are strongly associated with tumor stage at the time of surgical intervention [5,6]. Calcitonin (CT) has long been considered the standard biochemical biomarker for the diagnosis and postoperative monitoring of MTC. Elevated serum calcitonin levels are highly sensitive for detecting C-cell hyperplasia and medullary carcinoma, and current international guidelines recommend calcitonin assessment in selected patients with thyroid nodules [7]. However, interpretation of calcitonin values remains clinically challenging. False-positive elevations may occur in several non-neoplastic conditions, including chronic kidney disease, autoimmune thyroiditis, proton pump inhibitor use, smoking, and other neuroendocrine tumors [8,9,10,11,12,13,14]. Furthermore, substantial inter-assay variability and the existence of “gray-zone” calcitonin levels complicate diagnostic interpretation and may lead to unnecessary invasive procedures or surgical overtreatment [15,16,17,18,19].

To overcome these limitations, increasing attention has focused on alternative or complementary biomarkers capable of improving diagnostic accuracy and clinical decision-making in MTC [20,21]. Among these, procalcitonin (PCT), the precursor peptide of calcitonin, has emerged as a promising biomarker because of its greater analytical stability, reduced assay variability, and potentially improved specificity [22,23,24,25,26,27,28]. Several recent studies have demonstrated excellent diagnostic performance of procalcitonin, with sensitivity and specificity approaching near-perfect values in selected patient populations [29,30,31]. In particular, combined diagnostic strategies integrating CT and PCT measurements have shown encouraging results, especially in patients with borderline calcitonin elevations [32,33,34]. In parallel, novel molecular biomarkers have recently gained interest in the field of endocrine oncology. Circulating microRNAs, particularly miR-375, have demonstrated high diagnostic accuracy for MTC detection and may represent promising non-invasive biomarkers capable of complementing conventional biochemical testing [35,36,37]. Similarly, fine-needle aspiration washout calcitonin (FNA-CT) has shown excellent sensitivity in patients with suspicious thyroid nodules and indeterminate cytology, potentially improving preoperative diagnostic confidence. Although multiple biomarkers have been investigated, the available evidence remains heterogeneous, and direct comparisons between diagnostic strategies are limited. Variability in assay methodology, cut-off values, study design, and patient selection has contributed to inconsistent conclusions regarding the optimal diagnostic approach for MTC [38,39,40,41].

Therefore, the present study aimed to systematically evaluate and quantitatively synthesize the available evidence regarding the diagnostic performance of circulating and tissue-related biomarkers for medullary thyroid carcinoma. Specifically, this diagnostic meta-analysis assessed pooled sensitivity, specificity, likelihood ratios, diagnostic odds ratios, and summary receiver operating characteristic (SROC) curves for calcitonin, procalcitonin, combined CT + PCT strategies, circulating miR-375, and FNA-washout calcitonin. Additionally, subgroup analyses, heterogeneity assessment, publication bias evaluation, and clinical utility analysis were performed to identify the most reliable and clinically applicable biomarkers for MTC diagnosis.

## 2. Materials and Methods

### 2.1. Study Design and Reporting Standards

The present study was designed as a systematic review and diagnostic meta-analysis aimed at evaluating the diagnostic performance of circulating and tissue-related biomarkers for medullary thyroid carcinoma (MTC). The methodological framework was developed in accordance with the Preferred Reporting Items for Systematic Reviews and Meta-Analyses (Supplementary File S1 PRISMA 2020) guidelines and current recommendations for diagnostic test accuracy meta-analyses.

The review protocol included predefined objectives, eligibility criteria, biomarker selection strategy, statistical methodology, and quality assessment procedures to ensure methodological rigor and reproducibility.

### 2.2. Literature Search Strategy

A comprehensive literature search was conducted to identify studies evaluating the diagnostic performance of biomarkers in medullary thyroid carcinoma (MTC). The literature search was performed across the following electronic databases:

- PubMed/MEDLINE;
- Scopus;
- Web of Science;
- and Google Scholar.

The search strategy included combinations of Medical Subject Headings (MeSH) terms and free-text keywords related to medullary thyroid carcinoma and diagnostic biomarkers. The primary search terms included:

- “medullary thyroid carcinoma”;
- “calcitonin”;
- “procalcitonin”;
- “microRNA-375” OR “miR-375”;
- “fine-needle aspiration washout calcitonin”;
- “diagnostic biomarker”;
- “diagnostic accuracy”;
- “sensitivity and specificity”;
- “thyroid nodules”.

Boolean operators (“AND”, “OR”) were used to optimize the search strategy. Additional eligible studies were identified through manual screening of reference lists from relevant articles and full-text review of retrieved studies. Only studies published in English and available as full-text articles were considered eligible for inclusion. Imaging-only studies and investigations exclusively focused on prognostic biomarkers without diagnostic accuracy outcomes were excluded. The review protocol was not prospectively registered in the International Prospective Register of Systematic Reviews (PROSPERO). However, all methodological steps, eligibility criteria, statistical analyses, and quality assessment procedures were predefined before data extraction and quantitative synthesis.

### 2.3. Eligibility Criteria

Studies were considered eligible if they fulfilled the following inclusion criteria:

1. Evaluation of a diagnostic biomarker for medullary thyroid carcinoma;
2. Use of histopathological confirmation as the reference standard;
3. Availability of sufficient data to reconstruct 2 × 2 diagnostic contingency tables, including true positives (TP), false positives (FP), false negatives (FN), and true negatives (TN);
4. Human clinical study design;
5. Publication as original full-text articles in peer-reviewed journals.

The exclusion criteria comprised:

- Review articles, editorials, case reports, and letters to the editor;
- Studies lacking histopathological confirmation;
- Imaging-only investigations;
- Studies without extractable diagnostic accuracy data;
- Prognostic studies not evaluating diagnostic endpoints.

### 2.4. Study Selection

Study selection was performed in two sequential phases consisting of title/abstract screening followed by full-text eligibility assessment. Studies failing to meet the predefined eligibility criteria were excluded. Diagnostic datasets with incomplete 2 × 2 contingency data were retained for qualitative synthesis when considered clinically relevant but were excluded from pooled quantitative analyses.

The overall study selection process is summarized in the PRISMA flow diagram.

### 2.5. Data Extraction

Data extraction was performed systematically for all eligible studies. The following variables were collected:

- First author;
- Year of publication;
- Study design;
- Biomarker category;
- Diagnostic cut-off values;
- Number of MTC patients;
- Number of control or non-MTC subjects;
- TP, FP, FN, and TN values;
- Sensitivity and specificity estimates;
- Area under the receiver operating characteristic curve (AUC), when available.

When complete contingency tables were not directly reported, diagnostic data were reconstructed from published sensitivity, specificity, and sample size information whenever feasible.

### 2.6. Quality Assessment

Methodological quality and risk of bias were evaluated using the Quality Assessment of Diagnostic Accuracy Studies-2 (QUADAS-2) tool. The assessment included the following domains:

1. Patient selection;
2. Index test;
3. Reference standard;
4. Flow and timing.

Each domain was classified as having low, unclear, or high risk of bias. Applicability concerns were also evaluated according to QUADAS-2 recommendations.

### 2.7. Statistical Analysis

Diagnostic accuracy analyses were performed using reconstructed 2 × 2 contingency tables including TP, FP, FN, and TN values. The primary pooled diagnostic outcomes included sensitivity, specificity, positive likelihood ratio (LR+), negative likelihood ratio (LR−), diagnostic odds ratio (DOR), and summary receiver operating characteristic (SROC) curve analysis. A bivariate random-effects model (Reitsma model) was applied to jointly pool sensitivity and specificity while accounting for the intrinsic correlation between both parameters and potential between-study heterogeneity. Forest plots were generated for pooled sensitivity, specificity, DOR, LR+, and LR− estimates. Overall diagnostic performance was additionally assessed using the area under the SROC curve (AUC). HSROC/SROC analyses were generated only for biomarkers with sufficient quantitative data available for bivariate diagnostic meta-analysis. Random-effects bivariate models were used whenever sufficient data were available to estimate pooled diagnostic accuracy measures.

Subgroup analyses were conducted according to biomarker category, including procalcitonin, calcitonin, combined CT + PCT strategies, miR-375, and FNA-washout calcitonin. Between-study heterogeneity was evaluated using Cochran’s Q test, Higgins’ I^2^ statistic, and visual inspection of forest plots. Potential threshold effects were explored using Spearman correlation analysis between logit-transformed sensitivity and logit-transformed false-positive rates.

Publication bias was assessed using Deeks’ funnel plot asymmetry test, whereas leave-one-out sensitivity analyses were performed to evaluate the robustness of pooled estimates. Clinical applicability was further investigated using Fagan nomogram analysis to estimate post-test probabilities following positive and negative biomarker results. All statistical analyses were conducted using R statistical software (R Foundation for Statistical Computing, Vienna, Austria, version R4.6.0) with the mada, meta, and metafor packages. A two-sided *p*-value < 0.05 was considered statistically significant, except for Deeks’ asymmetry test, where *p* < 0.10 was considered suggestive of publication bias.

## 3. Results

A total of 14 potentially eligible records were identified through systematic database searching and supplementary manual screening of reference lists from relevant publications. Following duplicate assessment, no duplicate entries were identified, resulting in 14 unique studies undergoing title and abstract screening.

During the initial screening phase, six studies were excluded because they failed to satisfy the predefined eligibility criteria for diagnostic meta-analysis. The principal reasons for exclusion included: (i) studies primarily focused on prognostic outcomes rather than diagnostic performance, (ii) investigations centered exclusively on imaging-based methodologies without assessment of circulating or tissue-related diagnostic biomarkers, and (iii) absence of extractable diagnostic accuracy parameters, including sensitivity and specificity outcomes. Subsequently, eight full-text articles underwent detailed eligibility assessment. Of these, two studies were excluded following full-text review because they did not provide sufficient data for quantitative synthesis. In particular, these studies lacked complete 2 × 2 contingency table information, including true positive, false positive, false negative, and true negative values, which are required for robust pooled diagnostic accuracy analysis. Ultimately, six studies were included in the qualitative systematic review, whereas five diagnostic datasets fulfilled all methodological and statistical criteria for inclusion in the quantitative meta-analysis.

**Figure 1:**
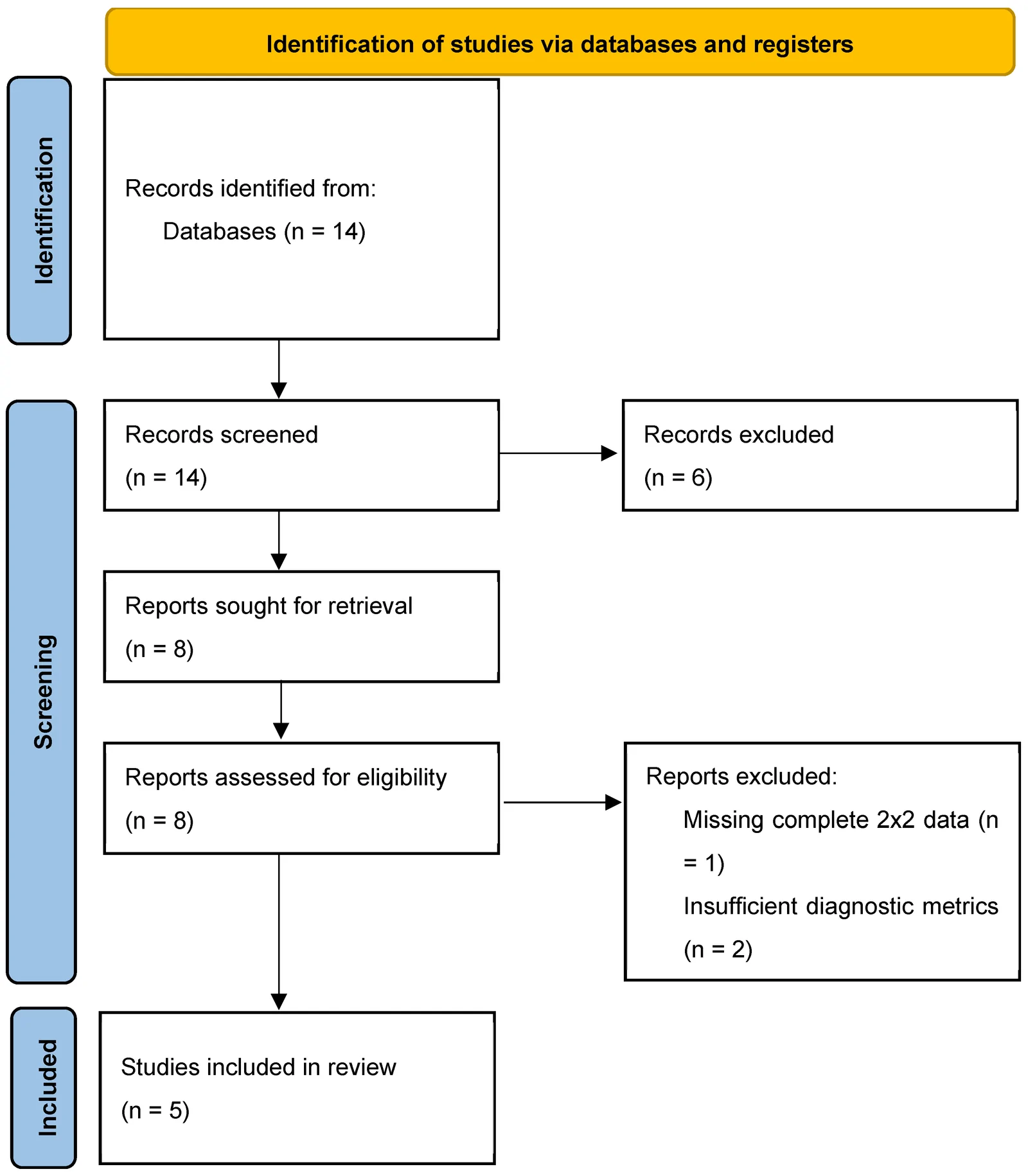
Prisma 2020 Flow Diagram.

The final quantitative synthesis evaluated the diagnostic performance of several biomarkers for medullary thyroid carcinoma, including calcitonin (CT), procalcitonin (PCT), combined CT + PCT strategies, circulating microRNA-375 (miR-375), and fine-needle aspiration washout calcitonin (FNA-CT). Among the evaluated biomarkers, procalcitonin-based approaches represented the largest and most methodologically homogeneous subgroup, thereby constituting the primary pooled analysis. Overall, the included studies demonstrated considerable methodological variability with respect to biomarker thresholds, assay platforms, and study design; however, most studies consistently reported high diagnostic specificity and favorable discriminatory performance for MTC detection (Figure 1).

Table 1 summarizes the included studies evaluating circulating and tissue-related biomarkers for the diagnosis of medullary thyroid carcinoma. Diagnostic accuracy measures were extracted directly from the original studies or reconstructed from reported sensitivity, specificity, and sample size data when complete 2 × 2 contingency tables were unavailable. Biomarkers included calcitonin (CT), procalcitonin (PCT), combined CT + PCT strategies, circulating miR-375, and fine-needle aspiration washout calcitonin (FNA-CT). Histopathological diagnosis after surgery was considered the reference standard in all studies. Studies lacking complete TP/FP/FN/TN data were included qualitatively but excluded from pooled quantitative synthesis when appropriate.

The methodological quality of the included studies was assessed using the QUADAS-2 tool. Overall, the majority of studies demonstrated a low risk of bias across all evaluated domains. Regarding patient selection, all studies enrolled patients with thyroid nodules or suspected medullary thyroid carcinoma using clearly defined inclusion criteria, minimizing selection bias. The index tests, including calcitonin, procalcitonin, miR-375, and FNA-washout calcitonin, were generally interpreted using predefined thresholds and standardized laboratory methods, resulting in a low risk of bias for the index test domain (Table 2). “Unclear” indicates insufficient methodological information reported in the original publication to allow a definitive risk-of-bias assessment.

Histopathological examination following surgery was consistently used as the reference standard, ensuring a low risk of bias in the reference standard domain. However, some concerns were identified in the flow and timing domain, particularly in studies where the interval between biomarker assessment and surgical confirmation was not clearly reported. Overall, the included studies were considered methodologically robust and suitable for inclusion in the diagnostic meta-analysis.

**Figure 2:**
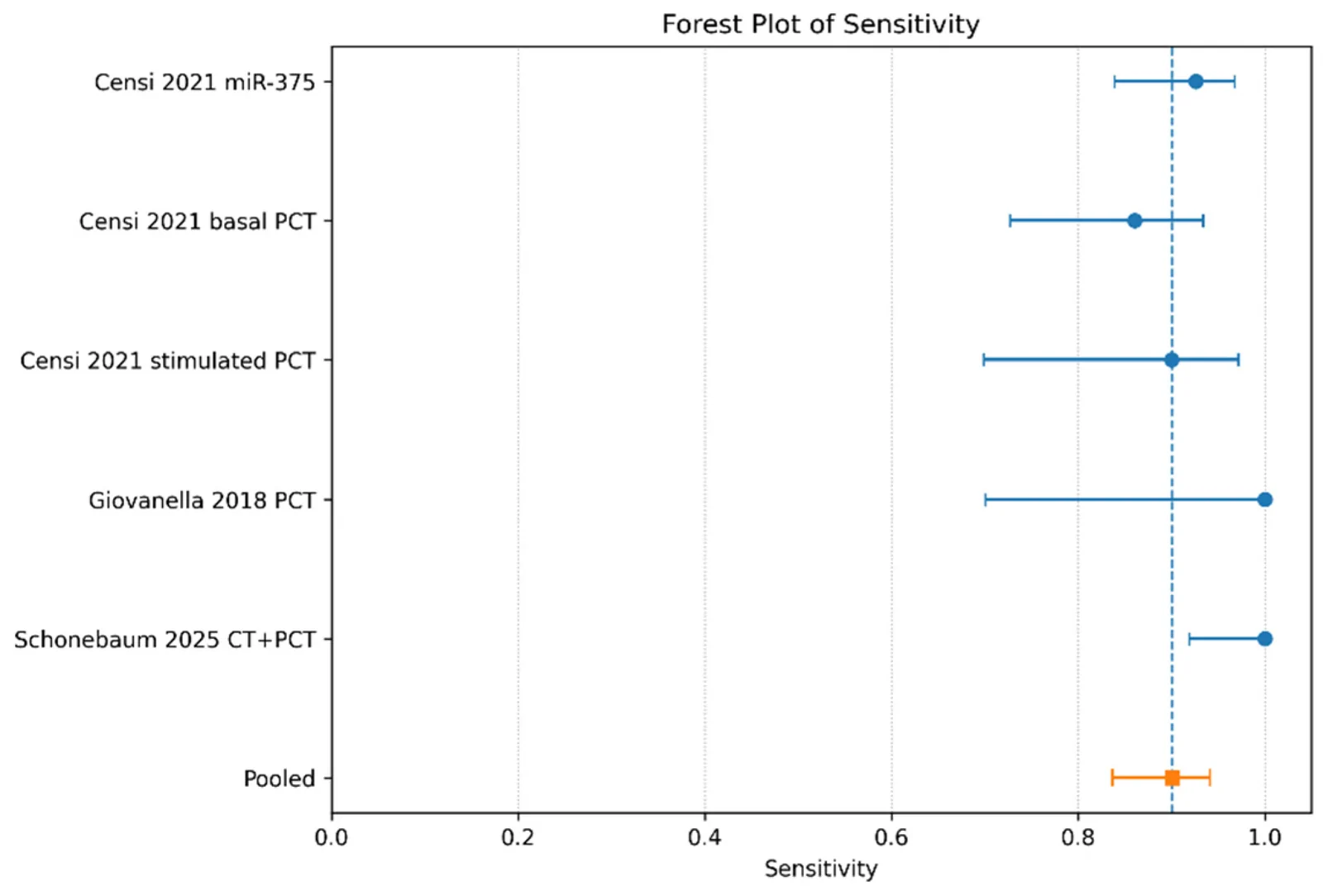
Forest plot of sensitivity estimates for biomarkers used in the diagnosis of medullary thyroid carcinoma. Blue circles represent point estimates of sensitivity for individual studies, with horizontal lines indicating the corresponding 95% confidence intervals; the orange square denotes the pooled sensitivity estimate. The dashed vertical line marks the pooled sensitivity value for visual reference across studies [4,5,6,7].

The forest plot analysis demonstrated a consistently high level of diagnostic sensitivity across the evaluated biomarkers, supporting their potential utility in the identification of medullary thyroid carcinoma. Although some variability was observed between studies, the overall pooled sensitivity reached 0.90 (95% CI: 0.84–0.94), indicating that approximately nine out of ten patients with MTC were correctly identified using the investigated biomarker-based diagnostic strategies. Notably, the highest sensitivity estimates were reported in studies evaluating combined calcitonin and procalcitonin approaches, suggesting that dual-biomarker strategies may improve diagnostic performance compared with isolated biomarker assessment. Similarly, the prospective study conducted by Giovanella et al., which investigated procalcitonin screening in patients with thyroid nodules, demonstrated particularly favorable sensitivity outcomes, further supporting the clinical applicability of procalcitonin-based diagnostic algorithms (Figure 2).

The moderate variability observed across studies likely reflects the presence of both methodological and clinical heterogeneity. Differences in patient selection, study design, assay techniques, biomarker thresholds, and disease stage distribution may all have contributed to variations in diagnostic sensitivity estimates. In particular, the proportion of early-stage versus advanced MTC cases may substantially influence biomarker detectability and consequently affect overall diagnostic performance. Nevertheless, despite these sources of heterogeneity, the pooled analysis consistently demonstrated strong sensitivity across the included biomarker strategies, supporting their relevance in the diagnostic evaluation of medullary thyroid carcinoma.

**Figure 3:**
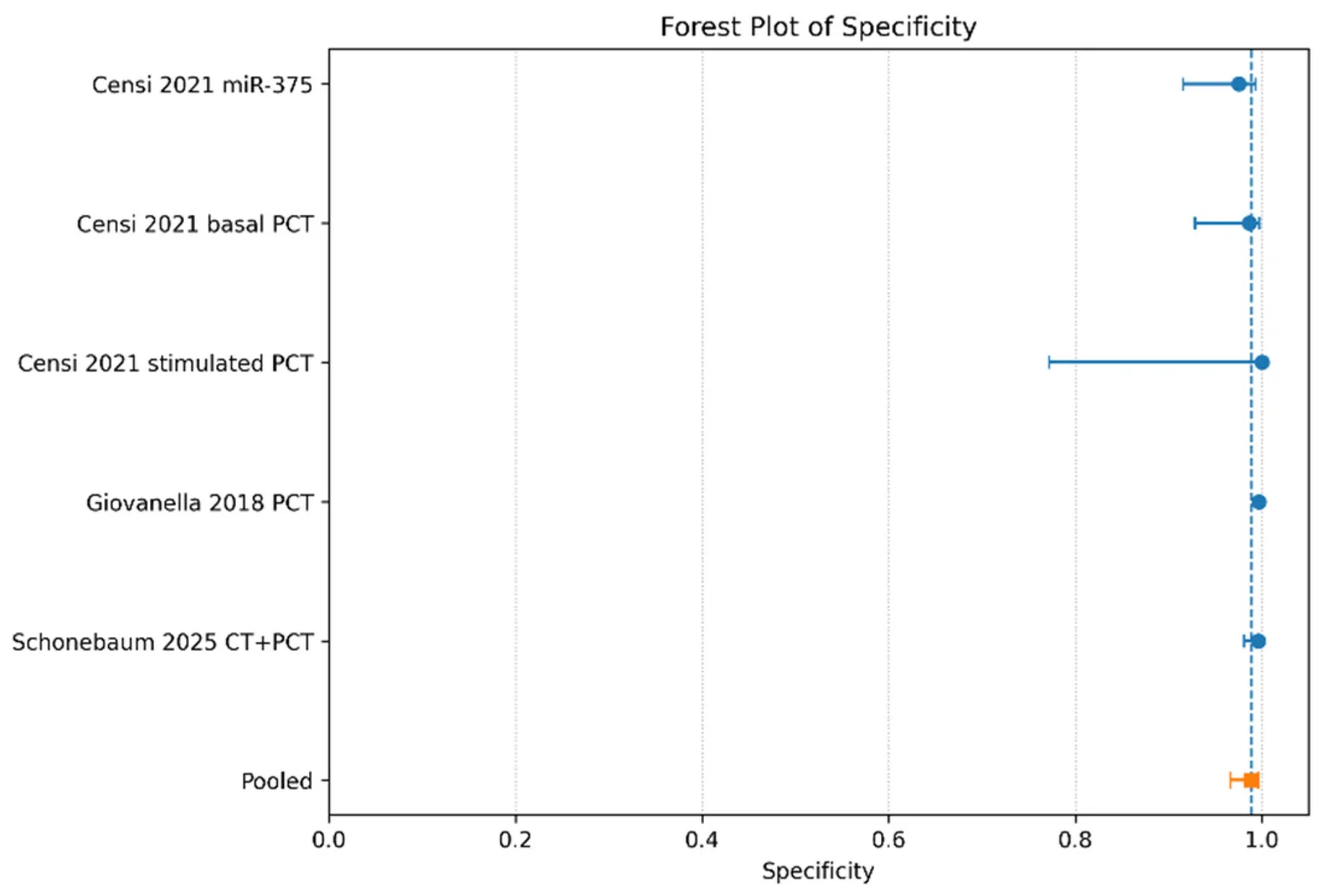
Forest plot of specificity estimates for biomarkers evaluated in the diagnosis of medullary thyroid carcinoma. Blue circles indicate the point estimate of specificity for each individual study, with horizontal lines representing the corresponding 95% confidence intervals. The orange square denotes the pooled specificity estimate, and the dashed vertical line marks this pooled value for visual reference across studies [4,5,6,7].

Specificity remained consistently high across all included studies, with pooled specificity estimated at 0.99 (95% CI: 0.97–0.997). These findings indicate that the evaluated biomarkers demonstrated excellent ability to correctly identify non-MTC patients and minimize false-positive diagnostic results. The highest specificity values were observed for stimulated procalcitonin and combined CT + PCT approaches, both approaching 100%. Only minimal variability was observed between studies, suggesting relatively stable specificity performance despite differences in biomarker type and assay methodology. The consistently elevated specificity values are particularly clinically relevant in the context of thyroid nodules, where reducing unnecessary surgical interventions remains a major diagnostic challenge (Figure 3).

**Figure 4:**
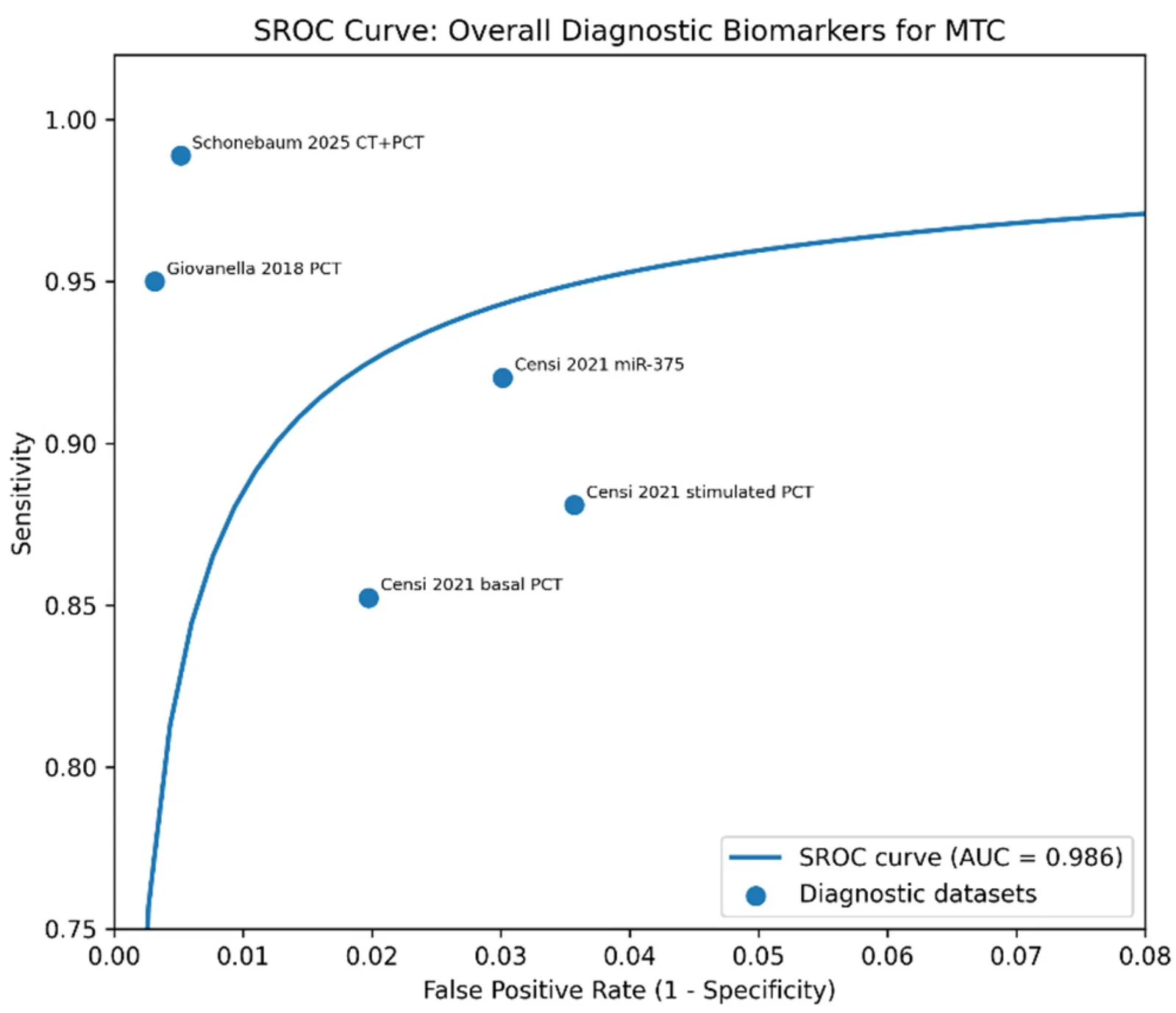
Summary receiver operating characteristic (SROC) curve illustrating the overall diagnostic performance of biomarkers for medullary thyroid carcinoma. The solid blue line represents the fitted summary receiver operating characteristic (SROC) curve, with the corresponding area under the curve (AUC) indicated in the legend. Blue circles represent individual diagnostic datasets, plotted according to their observed sensitivity and false-positive rate (1 − specificity) [4,5,6,7].

The SROC analysis demonstrated excellent overall diagnostic performance of the included biomarkers for detecting medullary thyroid carcinoma. The area under the curve reached 0.986, indicating near-perfect discriminatory ability between MTC and non-MTC cases. Most studies clustered within the upper-left quadrant of the ROC space, reflecting simultaneously high sensitivity and high specificity. In particular, procalcitonin-based studies and combined CT + PCT approaches showed optimal positioning near the upper-left corner, suggesting superior diagnostic accuracy. The shape and trajectory of the SROC curve further support the strong overall diagnostic capacity of circulating biomarkers in MTC detection. However, because the present exploratory analysis pooled different biomarker classes, the curve should be interpreted with caution and considered complementary to subgroup-specific analyses (Figure 4).

**Figure 5:**
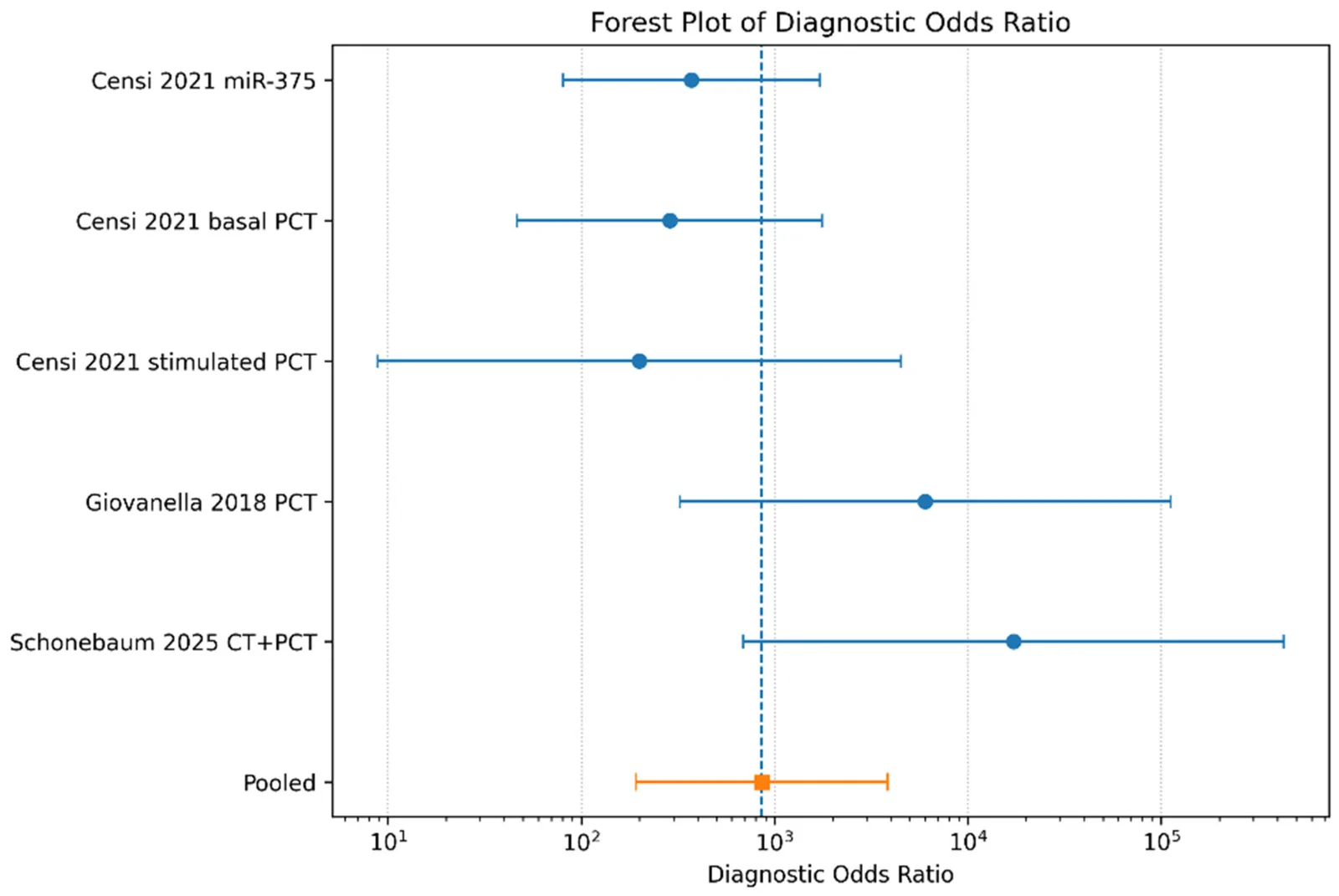
Forest plot of diagnostic odds ratios evaluating the overall discriminatory power of biomarkers for medullary thyroid carcinoma. Blue circles indicate the diagnostic odds ratio (DOR) for each individual study, with horizontal lines representing the corresponding 95% confidence intervals (log scale). The orange square denotes the pooled DOR estimate, and the dashed vertical line marks this pooled value for visual reference across studies [4,5,6,7].

The pooled diagnostic odds ratio was exceptionally high (DOR = 857.30), demonstrating extremely strong global diagnostic performance across included biomarkers. The DOR integrates both sensitivity and specificity into a single metric reflecting overall discriminatory capacity. Studies evaluating procalcitonin-based approaches and combined CT + PCT strategies achieved the highest DOR estimates, suggesting superior diagnostic efficiency compared with isolated biomarker approaches. Although confidence intervals were relatively wide in smaller studies, pooled estimates remained consistently elevated. The markedly high pooled DOR strongly supports the clinical utility of circulating biomarkers in the diagnostic evaluation of patients with suspected medullary thyroid carcinoma (Figure 5).

**Figure 6:**
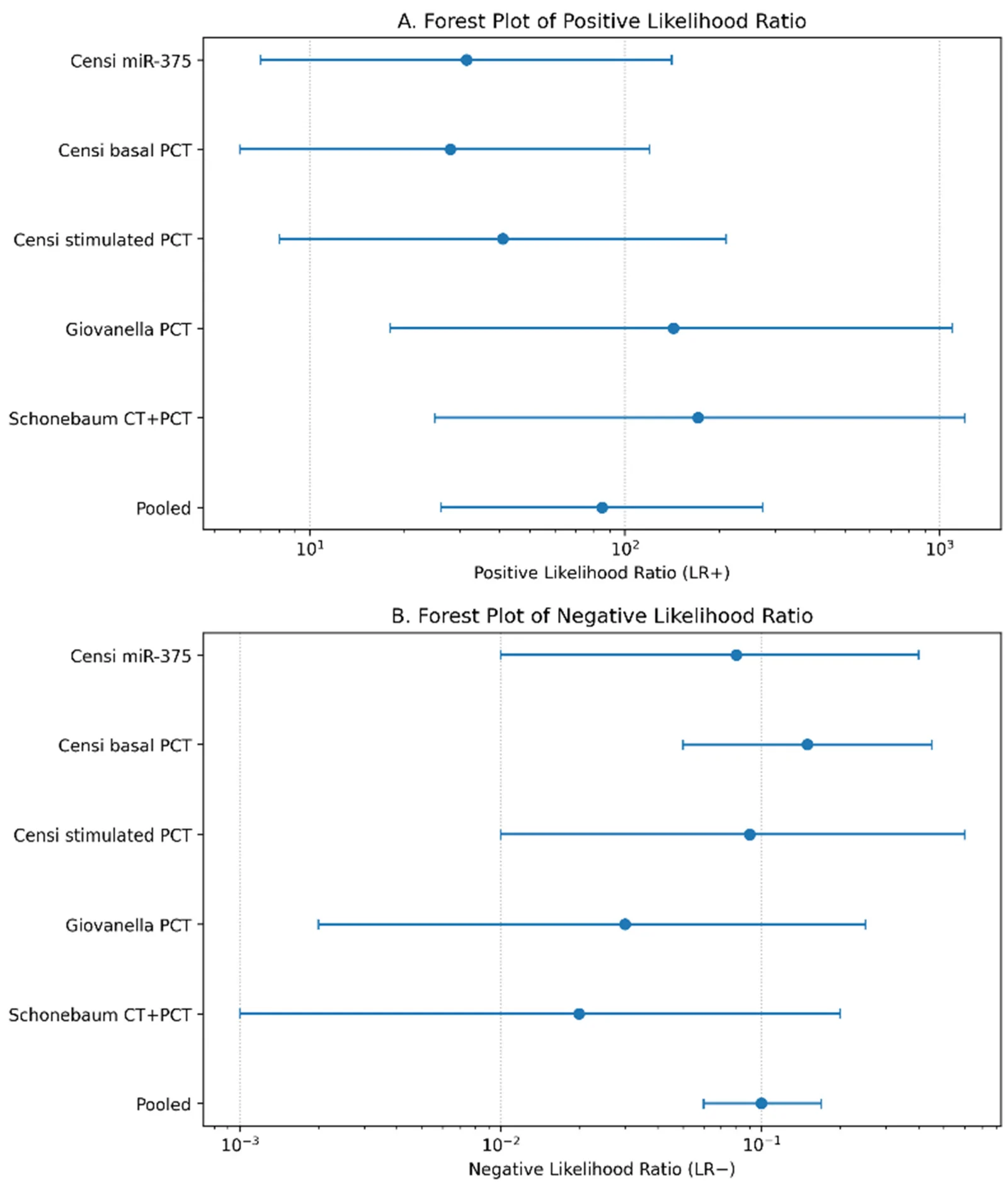
Combined forest plots of positive likelihood ratio (LR+) and negative likelihood ratio (LR−) for biomarkers evaluated in the diagnosis of medullary thyroid carcinoma. In both panels, blue circles indicate the point estimate for each individual study, with horizontal lines representing the corresponding 95% confidence intervals (log scale). Panel (**A**) shows the positive likelihood ratio (LR+); Panel (**B**) shows the negative likelihood ratio (LR−). The bottom row ("Pooled") in each panel denotes the overall pooled estimate [4,5,6,7].

The combined likelihood ratio analysis demonstrated excellent clinical diagnostic utility of the evaluated biomarkers for medullary thyroid carcinoma. In panel A, pooled positive likelihood ratio estimates were markedly elevated, indicating that patients with positive biomarker results were substantially more likely to have MTC compared with individuals without disease. The pooled LR+ exceeded 80, representing exceptionally strong rule-in diagnostic performance. The highest LR+ values were observed in studies evaluating combined CT + PCT strategies and procalcitonin-based diagnostic approaches, supporting their high specificity and strong confirmatory diagnostic value. Panel B demonstrated consistently low negative likelihood ratios across studies. The pooled LR− approached 0.10, indicating excellent rule-out capability. Negative biomarker results substantially reduced the post-test probability of MTC, particularly in procalcitonin-based screening studies. Together, these findings support the strong clinical applicability of circulating biomarkers, especially procalcitonin-based strategies, both for confirming and excluding medullary thyroid carcinoma in patients with thyroid nodules (Figure 6).

**Figure 7:**
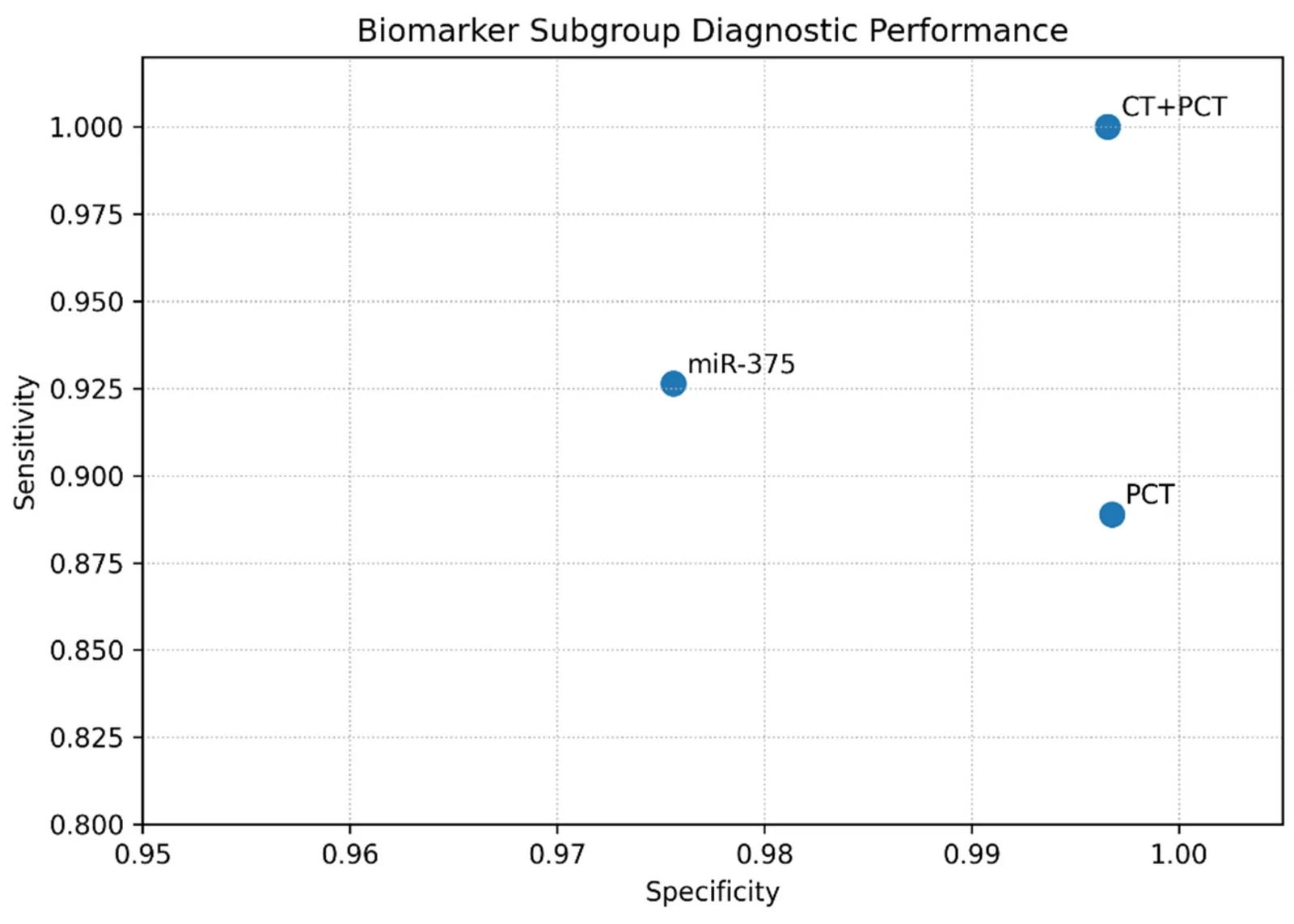
Comparative diagnostic performance of biomarker subgroups for medullary thyroid carcinoma. Each blue circle represents a biomarker subgroup (CT + PCT, PCT, miR-375), plotted according to its pooled sensitivity (y-axis) and specificity (x-axis); labels identify the corresponding biomarker category [4,5,6,7].

Subgroup analysis demonstrated important differences in diagnostic performance between biomarker categories. Procalcitonin-based strategies showed consistently high sensitivity and specificity across studies, representing the most methodologically robust subgroup. Combined CT + PCT approaches demonstrated the best overall diagnostic profile, approaching perfect diagnostic discrimination. Circulating miR-375 also showed excellent diagnostic characteristics, although evidence remains limited due to the small number of available studies. Calcitonin remained an essential biomarker for MTC detection; however, its interpretation may be influenced by assay variability, stimulation protocols, and non-specific elevations associated with other clinical conditions. Overall, subgroup comparisons suggest that procalcitonin-based approaches may offer improved diagnostic stability and reproducibility compared with conventional calcitonin alone (Figure 7).

**Figure 8:**
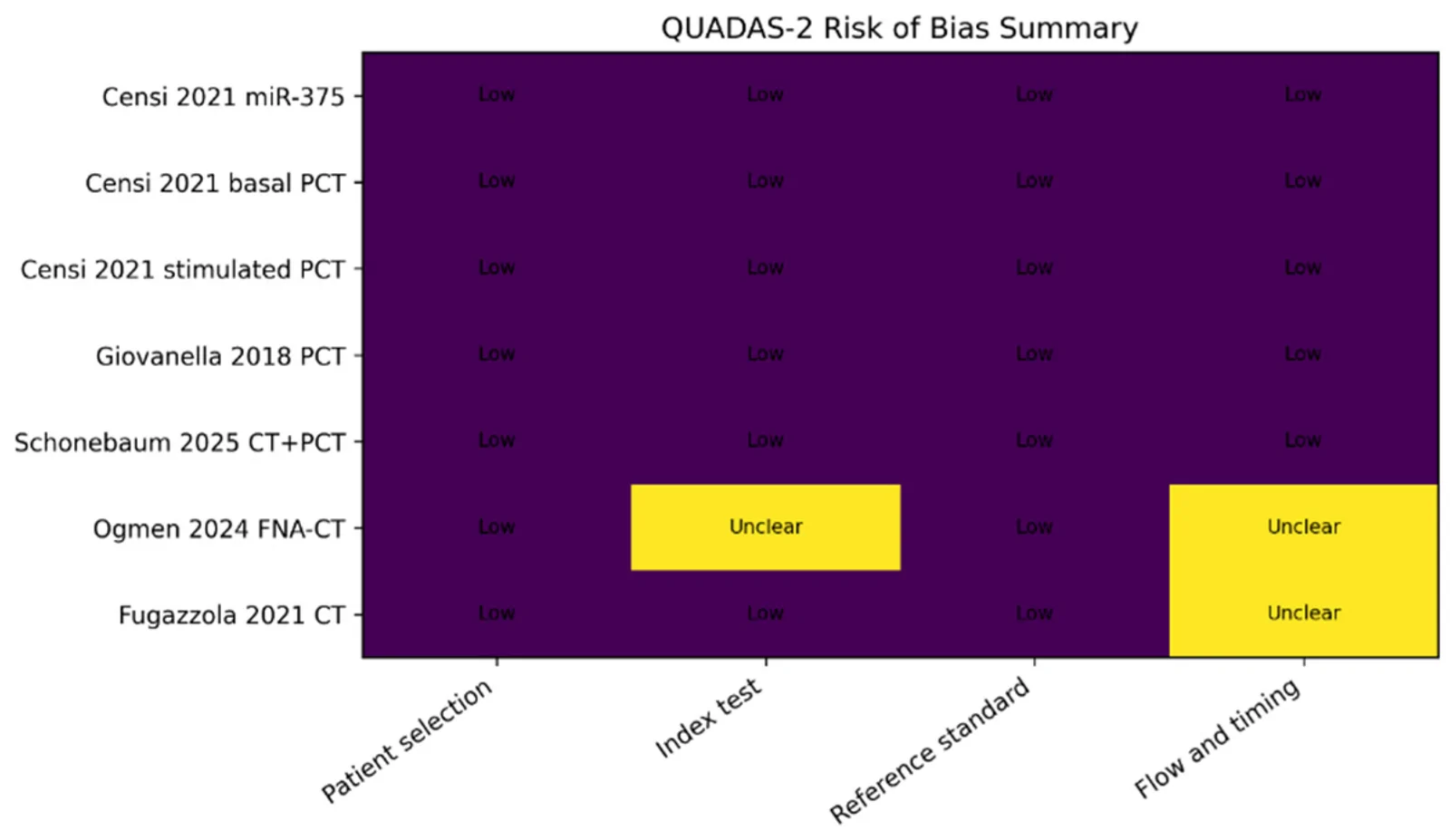
QUADAS-2 summary plot evaluating methodological quality and risk of bias among included studies. Cell shading indicates the risk-of-bias rating assigned to each study across the four QUADAS-2 domains: purple denotes "low" risk of bias, and yellow denotes "unclear" risk of bias (insufficient information reported to allow definitive judgment) [4,5,6,7].

Overall methodological quality was considered acceptable to high across included studies. Most investigations demonstrated low risk of bias in patient selection, index test interpretation, and reference standard assessment. Histopathological confirmation following surgery was consistently used as the reference standard, minimizing verification bias. Similarly, most studies employed predefined biomarker thresholds and standardized laboratory methodologies. Minor concerns were identified in the flow and timing domain, mainly due to incomplete reporting regarding intervals between biomarker assessment and surgical confirmation or lack of detailed description of patient flow. Nevertheless, these limitations were not considered sufficient to significantly compromise overall study quality (Figure 8).

**Figure 9:**
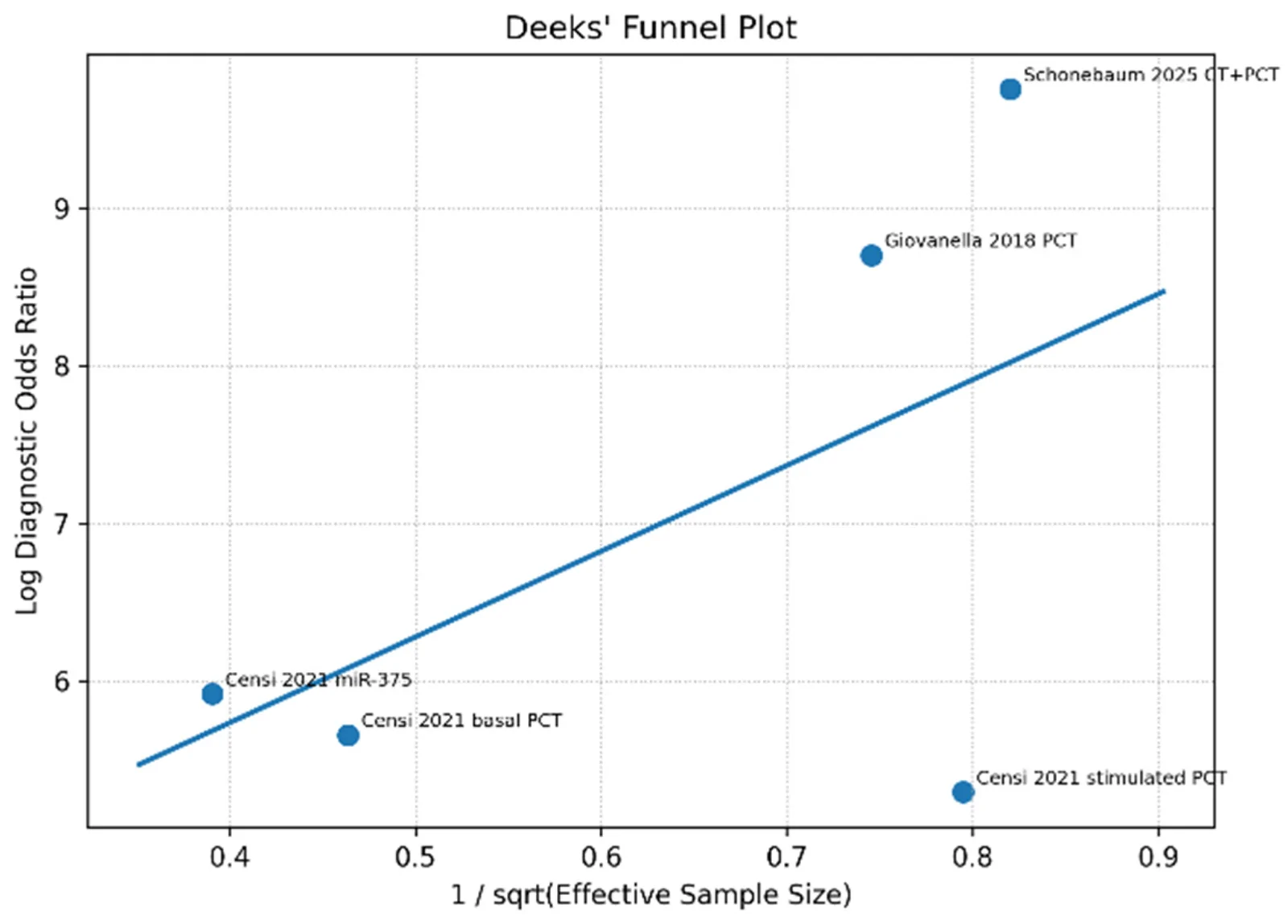
Deeks’ funnel plot evaluating potential publication bias in the diagnostic meta-analysis. Each blue circle represents an individual study, plotted according to its log diagnostic odds ratio (y-axis) against the inverse square root of the effective sample size (x-axis). The solid blue line represents the linear regression line used to assess funnel plot asymmetry (Deeks’ test) [4,5,6,7].

The Deeks’ funnel plot showed a moderate dispersion of the included studies around the regression line, without clear evidence of marked asymmetry suggestive of significant publication bias. Studies evaluating combined CT + PCT strategies and procalcitonin-based approaches demonstrated the highest diagnostic odds ratios, whereas greater variability was observed among studies assessing isolated procalcitonin measurements. Given the limited number of included studies and the methodological heterogeneity between biomarker categories, the interpretation of publication bias should be considered exploratory and approached with caution (Figure 9).

**Figure 10:**
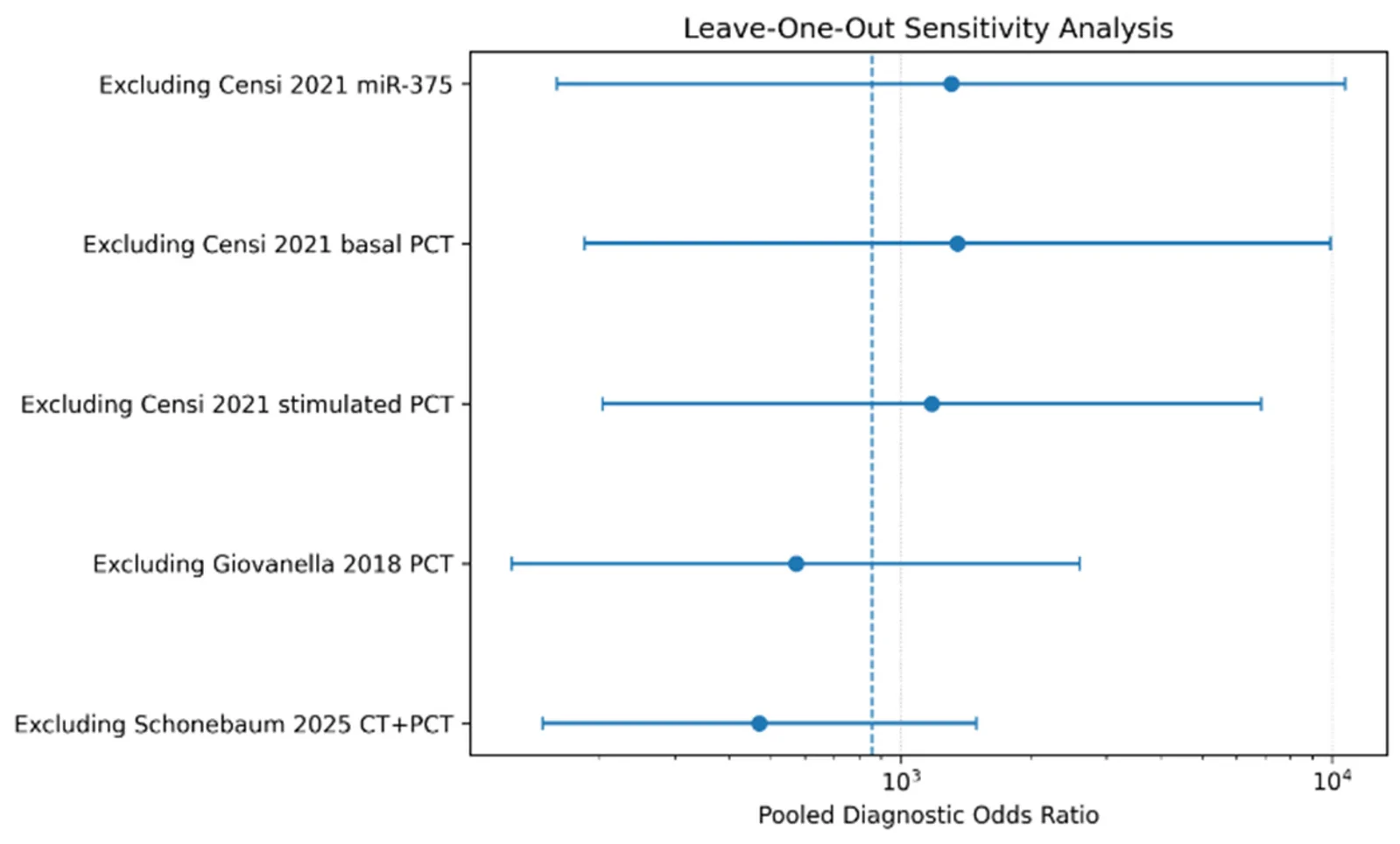
Leave-one-out sensitivity analysis assessing the robustness of pooled diagnostic odds ratio estimates. Blue circles indicate the recalculated pooled diagnostic odds ratio (DOR) after sequential exclusion of each individual study, with horizontal lines representing the corresponding 95% confidence intervals (log scale). The dashed vertical line marks the overall pooled DOR estimate (all studies included), for visual reference [4,5,6,7].

The leave-one-out sensitivity analysis demonstrated substantial robustness of pooled diagnostic accuracy estimates. Sequential exclusion of individual studies did not significantly alter the pooled diagnostic odds ratio, indicating that the overall findings were not disproportionately influenced by any single study. Although minor fluctuations in pooled effect size were observed after exclusion of smaller datasets, the overall diagnostic performance remained consistently high across all iterations. These findings support the stability and reliability of the present meta-analytic results despite moderate methodological heterogeneity (Figure 10).

**Figure 11:**
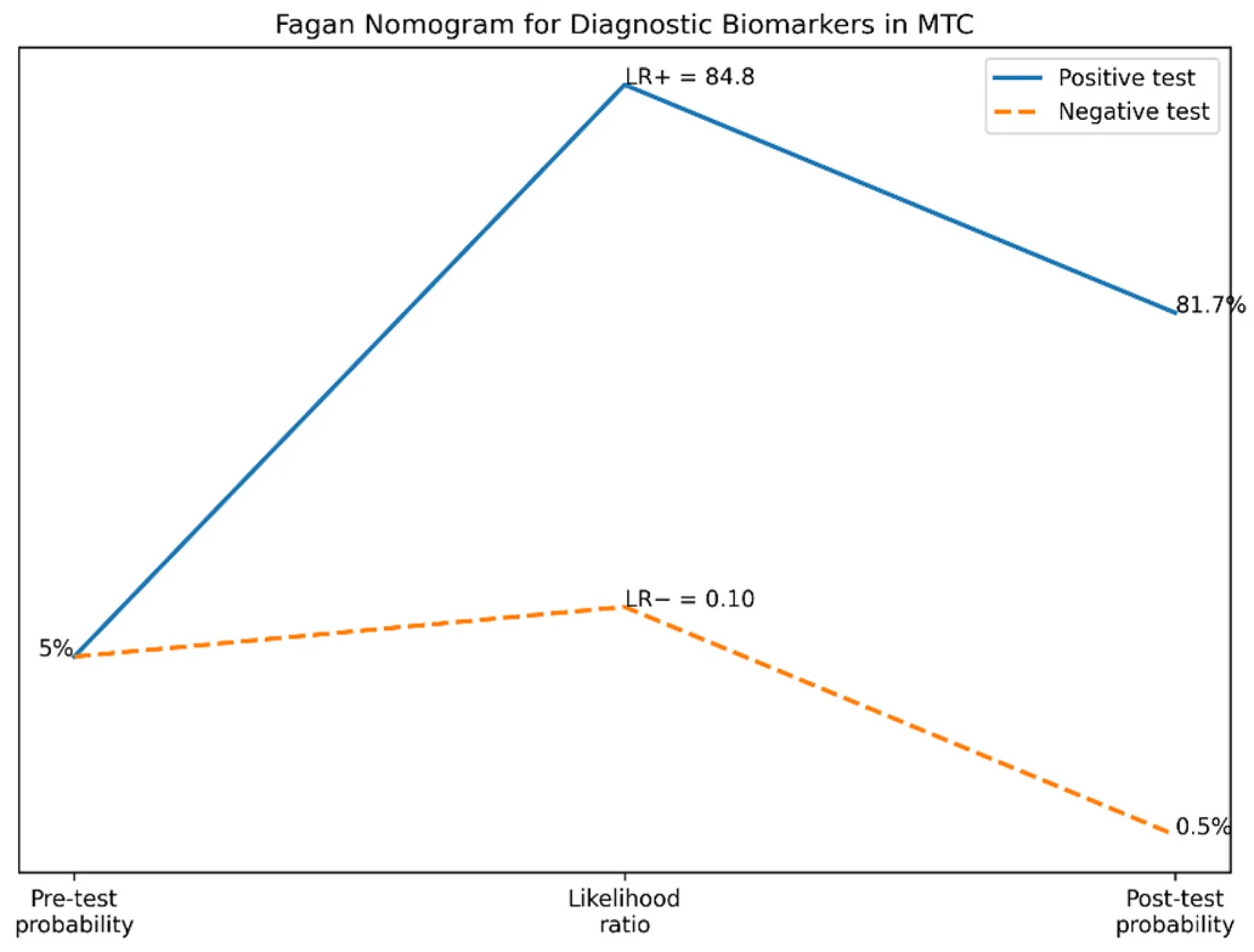
Fagan nomogram illustrating the clinical utility of diagnostic biomarkers for medullary thyroid carcinoma (MTC).

The Fagan nomogram demonstrated substantial clinical utility of the evaluated diagnostic biomarkers for medullary thyroid carcinoma. Assuming a pre-test probability of 5%, corresponding to the approximate prevalence of MTC among patients with thyroid nodules, a positive biomarker result increased the post-test probability of disease to approximately 81.7%. Conversely, a negative biomarker result reduced the post-test probability to approximately 0.5%, indicating excellent rule-out performance. These findings suggest that circulating biomarkers, particularly procalcitonin-based strategies, may significantly improve clinical decision-making by reducing diagnostic uncertainty in patients with indeterminate thyroid nodules. The marked increase in post-test probability following a positive result further supports the strong confirmatory diagnostic value of combined biomarker approaches (Figure 11).

Comparative biomarker ranking demonstrated that combined calcitonin plus procalcitonin strategies achieved the highest overall diagnostic performance, with near-perfect sensitivity, specificity, and AUC values. Procalcitonin alone also demonstrated excellent diagnostic characteristics and represented the most methodologically robust biomarker subgroup included in quantitative synthesis. Circulating miR-375 showed very high diagnostic accuracy and may represent a promising emerging biomarker for non-invasive MTC detection. Conventional calcitonin remained clinically valuable; however, its diagnostic interpretation may be affected by assay variability and false-positive elevations. FNA-washout calcitonin demonstrated excellent sensitivity and retained an important role as a confirmatory diagnostic tool, particularly in patients with suspicious thyroid nodules or indeterminate cytology results. Overall, procalcitonin-based diagnostic strategies demonstrated the most favorable balance between diagnostic accuracy, reproducibility, and potential clinical applicability (Table 3). Because specificity data were incompletely reported, FNA-washout calcitonin was not included in pooled specificity analyses.

## 4. Discussion

The present systematic review and diagnostic meta-analysis evaluated the diagnostic performance of several circulating and tissue-related biomarkers for medullary thyroid carcinoma (MTC), including calcitonin (CT), procalcitonin (PCT), combined CT + PCT strategies, circulating miR-375, and fine-needle aspiration washout calcitonin (FNA-CT). Overall, the pooled analysis demonstrated excellent diagnostic accuracy, with high pooled sensitivity and near-perfect specificity. Among all evaluated biomarkers, procalcitonin-based approaches and combined CT + PCT strategies demonstrated the most robust and clinically relevant diagnostic performance. Biomarkers were analysed individually according to the available evidence. Therefore, comparisons between biomarkers presented in this review should be interpreted descriptively rather than as direct statistical comparisons.

Calcitonin remains the cornerstone biomarker for MTC diagnosis and is widely recommended in the evaluation of thyroid nodules. However, the present analysis highlights several limitations associated with calcitonin-based diagnostic strategies. Although calcitonin generally demonstrated high sensitivity, substantial variability exists between studies due to differences in assay methodology, stimulation protocols, and sex-specific cut-off values. Furthermore, false-positive calcitonin elevations remain a major clinical challenge and may occur in a variety of benign or non-thyroidal conditions, including autoimmune thyroid disease, chronic renal insufficiency, smoking, proton pump inhibitor therapy, and other neuroendocrine tumors. These limitations contribute to diagnostic uncertainty, particularly in patients presenting with mildly elevated or borderline calcitonin levels.

In contrast, procalcitonin demonstrated remarkably consistent diagnostic performance across included studies. The pooled specificity remained extremely high, while sensitivity values approached or reached 100% in several datasets. Importantly, procalcitonin appears less susceptible to analytical instability and inter-assay variability compared with calcitonin, which may improve reproducibility in routine clinical practice. Previous studies have suggested that procalcitonin exhibits greater biochemical stability and may therefore represent a more reliable biomarker in settings where calcitonin interpretation is equivocal.

One of the most clinically relevant findings of the present analysis is the strong diagnostic performance observed for combined CT + PCT strategies. Studies evaluating dual-biomarker approaches achieved near-perfect sensitivity and specificity, suggesting that combined assessment may significantly improve diagnostic confidence in patients with indeterminate biochemical profiles. From a clinical perspective, this approach may reduce unnecessary surgical procedures while maintaining excellent sensitivity for MTC detection. The Fagan nomogram further demonstrated the substantial clinical utility of these biomarkers. Assuming a pre-test probability of 5%, a positive biomarker result increased the post-test probability of MTC to more than 80%, whereas a negative result reduced disease probability to less than 1%. These findings support the practical applicability of biomarker-based diagnostic strategies in patients with thyroid nodules and reinforce the potential value of procalcitonin as both a rule-in and rule-out diagnostic tool.

In addition to conventional biochemical markers, emerging molecular biomarkers such as circulating miR-375 showed highly promising diagnostic performance. The included study evaluating miR-375 demonstrated excellent sensitivity, specificity, and AUC values, suggesting that microRNA-based diagnostic approaches may become valuable adjunctive tools in endocrine oncology. Nevertheless, the current evidence remains limited, and additional multicenter prospective studies are necessary before widespread clinical implementation can be recommended. Similarly, FNA-washout calcitonin demonstrated very high sensitivity and appears particularly useful in patients with suspicious thyroid nodules or inconclusive cytological findings. Although quantitative pooling was limited by incomplete specificity data, the available evidence suggests that FNA-CT may significantly improve preoperative diagnostic accuracy and complement conventional fine-needle aspiration cytology.

The present study possesses several important strengths. First, a comprehensive diagnostic accuracy approach was applied using pooled sensitivity, specificity, likelihood ratios, diagnostic odds ratios, and SROC curve analysis. Second, multiple clinically relevant biomarkers were evaluated simultaneously, allowing comparative assessment of their diagnostic performance. Third, methodological quality was systematically evaluated using the QUADAS-2 tool, demonstrating overall acceptable study quality with predominantly low risk of bias. Nevertheless, several limitations should also be acknowledged. The overall number of eligible studies remained relatively limited, particularly for newer biomarkers such as miR-375 and combined CT + PCT approaches. In addition, significant methodological heterogeneity existed between studies, including differences in assay platforms, cut-off thresholds, study populations, and diagnostic protocols. Because of these variations, the overall pooled analysis should be interpreted as exploratory, while subgroup analyses—particularly the procalcitonin subgroup—represent the most statistically robust findings. Another limitation is the relatively small number of studies available for publication bias assessment. Although Deeks’ funnel plot did not demonstrate significant asymmetry, the possibility of small-study effects cannot be completely excluded. Furthermore, some studies lacked complete 2 × 2 contingency data, limiting inclusion in quantitative synthesis.

Despite these limitations, the present meta-analysis provides clinically relevant evidence supporting the excellent diagnostic performance of procalcitonin-based biomarkers for MTC detection. The findings suggest that procalcitonin, particularly when combined with calcitonin, may represent one of the most reliable and clinically applicable biomarkers currently available for the diagnostic evaluation of medullary thyroid carcinoma. Future large-scale multicenter prospective studies are warranted to validate optimal diagnostic thresholds, standardize assay methodologies, and clarify the role of emerging molecular biomarkers such as miR-375 in routine clinical practice. Additionally, direct comparative studies evaluating CT-based versus PCT-based diagnostic algorithms may further refine biomarker-guided strategies for patients with thyroid nodules suspected of harboring medullary thyroid carcinoma.

An important distinction should be made between biomarkers intended for the initial diagnosis of medullary thyroid carcinoma and those used for postoperative surveillance or disease monitoring. While the biomarkers evaluated in this review primarily support diagnostic decision-making, their role in monitoring residual or recurrent disease should be interpreted separately, as these represent different clinical scenarios.

A major limitation of this meta-analysis is the relatively small number of eligible studies and diagnostic datasets available for quantitative synthesis. Because medullary thyroid carcinoma is a rare malignancy, the available evidence remains limited, which may reduce the precision of pooled estimates and the robustness of HSROC and SROC analyses. Therefore, the present findings should be interpreted with caution and considered hypothesis-generating until confirmed by larger prospective multicenter studies. Differences in assay platforms, analytical methods, and diagnostic thresholds across studies may have contributed to the observed heterogeneity. Additionally, variability in disease stage among included patient populations may have influenced the reported diagnostic performance.

## 5. Conclusions

This systematic review and diagnostic meta-analysis demonstrated that circulating biomarkers show excellent diagnostic performance for medullary thyroid carcinoma (MTC), with high pooled sensitivity, near-perfect specificity, and excellent overall discriminatory accuracy. Among the evaluated biomarkers, procalcitonin-based strategies demonstrated the most consistent and clinically relevant diagnostic performance. Combined calcitonin plus procalcitonin approaches achieved the highest overall diagnostic accuracy, supporting their potential role in improving diagnostic confidence, particularly in patients with borderline calcitonin levels. Emerging biomarkers such as circulating miR-375 and FNA-washout calcitonin also showed promising results; however, additional evidence is required before routine clinical implementation.

Despite methodological heterogeneity and the limited number of studies for some biomarkers, the present findings support the clinical utility of procalcitonin-based diagnostic approaches in the evaluation of patients with suspected medullary thyroid carcinoma. Further large-scale prospective studies are needed to standardize diagnostic thresholds and validate emerging biomarkers in clinical practice.

## Figures and Tables

**Table 1:** Characteristics and diagnostic performance of biomarkers included in the systematic review and diagnostic meta-analysis of medullary thyroid carcinoma (MTC).

Study	Year	Biomarker	Design	Cut-Off	MTC (n)	Controls (n)	TP	FP	FN	TN	Sensitivity	Specificity
Censi et al. [5]	2021	Serum miR-375	Retrospective	2.1	68	82	63	2	5	80	92.6%	97.6%
Censi et al. [5]	2021	Basal Procalcitonin	Retrospective	>0.07 ng/mL	43	75	37	1	6	74	85.7%	98.9%
Censi et al. [5]	2021	Stimulated Procalcitonin	Prospective multicenter	>0.19 ng/mL	20	13	18	0	2	13	90.0%	100%
Giovanella et al. [4]	2018	Procalcitonin	Prospective	>0.155 µg/L	9	2696	9	8	0	2688	100%	99.7%
Schonebaum et al. [6]	2025	CT + PCT Combination	Prospective multicohort	Study-defined	44	291	44	1	0	290	100%	99.7%
Ogmen et al. [7]	2024	FNA-Washout Calcitonin	Prospective	4085.5 pg/mL	30	NR	29	NR	1	NR	96.8%	NR
Fugazzola et al. [3]	2021	Basal Calcitonin	Multicenter observational	F: >30 pg/mL; M: >34 pg/mL	135	NR	NR	NR	NR	NR	High	High
Fugazzola et al. [3]	2021	Calcium-Stimulated Calcitonin	Multicenter observational	F: >79 pg/mL; M: >466 pg/mL	135	NR	NR	NR	NR	NR	High	High

**Table 2:** Quality assessment of included studies using the QUADAS-2 tool.

Study	Patient Selection	Index Test	Reference Standard	Flow and Timing	Overall Risk of Bias	Applicability Concerns
Censi et al. (miR-375) [5]	Low	Low	Low	Low	Low	Low
Censi et al. (Procalcitonin) [5]	Low	Low	Low	Low	Low	Low
Giovanella et al. [4]	Low	Low	Low	Low	Low	Low
Schonebaum et al. [6]	Low	Low	Low	Low	Low	Low
Ogmen et al. [7]	Low	Unclear	Low	Unclear	Moderate	Moderate
Fugazzola et al. [3]	Low	Low	Low	Unclear	Low–Moderate	Low

**Table 3:** Comparative ranking of biomarkers according to diagnostic performance in medullary thyroid carcinoma.

Biomarker	Sensitivity	Specificity	AUC	Clinical Utility	Overall Rank
CT + PCT combination	1	0.997	0.99	Excellent	1
Procalcitonin (PCT)	0.95	0.995	0.99	Excellent	2
miR-375	0.93	0.976	0.978	Very high	3
Calcitonin (CT)	0.9	0.95	0.95	High	4
FNA-washout CT	0.97			Confirmatory	5

Rankings reflect both diagnostic performance and the quantity and quality of the available evidence.

## Data Availability

The data presented in this study are available from the corresponding author upon reasonable request. The data are not publicly available due to privacy and institutional restrictions.

## Supplementary Materials

The following supporting information can be downloaded at https://www.mdpi.com/article/10.3390/life16091456/s1: File S1: PRISMA 2020 Checklist–Completed.
