# Supplementary material for: Diagnostic Accuracy of Calcitonin, Procalcitonin, proGRP, miR-375 and FNA-Washout Calcitonin for Medullary Thyroid Carcinoma: A Systematic Review and Diagnostic Meta-Analysis
Source: Life (Basel). 2026 Aug 31;16(9):1456. doi: 10.3390/life16091456 (PMC13608150; doi:10.3390/life16091456)
Supplement: Supplementary file 1 [file life-16-01456-s001.zip › life-4366211-supplementary.pdf]

File S1: PRISMA 2020 Checklist – Completed

PRISMA Item	Location in Manuscript
1. Title	Page 1
2. Abstract	Pages 1–2
3. Rationale	Pages 2–3
4. Objectives	Page 3
5. Eligibility criteria	Pages 4–5
6. Information sources	Page 4
7. Search strategy	Page 4
8. Selection process	Page 5
9. Data collection process	Page 5
10a. Data items (outcomes)	Pages 5–6
10b. Other variables	Page 5
11. Study risk of bias assessment	Pages 5–6
12. Effect measures	Page 6
13a. Eligibility for synthesis	Pages 5–6
13b. Data preparation methods	Page 5
13c. Methods for tabulation/display	Pages 6–16
13d. Synthesis methods/meta-analysis model	Page 6
13e. Heterogeneity analyses	Page 6
13f. Sensitivity analyses	Pages 6, 16
14. Reporting bias assessment	Pages 6, 15
15. Certainty assessment	Not performed
16a. Study selection results	Pages 6–7
16b. Excluded studies/reasons	Pages 6–7
17. Study characteristics	Pages 8–9
18. Risk of bias in studies	Pages 9, 15
19. Results of individual studies	Pages 8–17
20a. Summary of contributing studies	Pages 8–9
20b. Statistical synthesis results	Pages 10–17
20c. Heterogeneity investigations	Pages 10, 12, 19
20d. Sensitivity analyses	Page 16
21. Reporting biases	Page 15
22. Certainty of evidence	Not performed
23a. Interpretation of results	Pages 17–19
23b. Limitations of evidence	Page 19
23c. Limitations of review process	Page 19
23d. Implications for practice/future research	Pages 18–19
24a. Registration information	Page 4
24b. Protocol access	Page 4
24c. Amendments to protocol	Not applicable
25. Support	Page 20
26. Competing interests	Page 20
27. Availability of data/materials	Page 20

Prepared according to the uploaded manuscript and PRISMA 2020 checklist.
